# Exploring the mechanisms underlying the last male precedence in the North African houbara bustard

**DOI:** 10.1242/jeb.251565

**Published:** 2025-12-19

**Authors:** Léna Meunier, Gabriele Sorci, Caroline Silva Vieira, Sadiya Sadiq Shiek, Yves Hingrat, Michel Saint Jalme, Janaina Torres Carreira

**Affiliations:** ^1^ Reneco International Wildlife Consultants LTD, Research department, Abu Dhabi 00000, United Arab Emirates; ^2^ Biogéosciences, UMR 6282 CNRS, Université de Bourgogne, Dijon 21000, France; ^3^ Federal University of Minas Gerais, UFMG, Department of Veterinary Clinical and Surgical Medicine, 31270-901, Belo Horizonte-MG, Brazil

**Keywords:** Sperm competition, Last male precedence, Sperm storage tubules, *Chlamydotis undulata*

## Abstract

The last male precedence (LMP) effect refers to the competitive advantage of the last male in the mating sequence. We conducted *in vitro* and *in vivo* experiments to explore the mechanisms underlying LMP in the North African houbara bustard (*Chlamydotis undulata*). Ejaculates were stained with two nuclear fluorescent stains [Hoechst 33342 (HBlue) and NUCLEAR-ID^®^ Red DNA (NRed)] and a mitochondrial stain [MitoTracker™ Red FM (MRed)], and motility and velocity of stained and unstained sperm were compared. Given that NRed impaired sperm motility and velocity, HBlue and MRed were retained for *in vivo* experiments. In a first *in vivo* experiment, females were inseminated with HBlue-stained sperm and the sperm stored in tubules were counted at 24, 48 and 72 h post-insemination. The number of sperm counted at the three timings was not significantly different, suggesting no sperm loss over 72 h post-insemination. In a second *in vivo* experiment, we performed sequential inseminations where females were either first inseminated with HBlue-stained sperm and subsequently with MRed-stained sperm, or vice versa. Twenty-four hours after the second insemination, we counted the number of stored sperm and assessed their location within the tubules and their distribution across the tubules. The majority of tubules (79.1%) contained both HBlue and MRed sperm. However, tubules contained more sperm from the second insemination, and they were located significantly closer to the bottom of the tubule. We discuss these results in light of the possible mechanisms underlying the LMP.

## Teaser

**Summary:** Nuclear and mitochondrial fluorescent stains reveal that sperm displacement during successive matings likely plays a role in explaining the last male precedence effect in the North African houbara bustard.

## INTRODUCTION

In many species, females have the ability to store sperm inside their reproductive tract, allowing fertilization of an egg long after mating (Bakst et al., 1994; Holt and Fazeli, 2016). In all bird species where this phenomenon has been investigated, sperm are stored inside specialised structures located inside mucosa folds of the utero–vaginal junction (UVJ), called sperm storage tubules (SSTs) (Blesbois and Brillard, 2007). The duration of sperm storage and the number of SSTs vary among species (Birkhead and Møller, 1992). For instance, ring doves (*Streptopelia risoria*) can store sperm up to 6 days (Zenone et al., 1979), Japanese quails (*Coturnix japonica*) up to 12 days (Reddish et al., 1996) while storage can extend up to 72 days in turkeys (*Meleagris gallopavo*) (Lorenz, 1950). The number of SSTs ranges from a few hundred in passerines (Briskie, 1996a), 2000–3000 in the chicken (*Gallus domesticus*) (Brillard et al., 1998) and up to 20,000 in the turkey (Goodrich-Smith and Marquez, 1978). When females mate with multiple males, a post-copulatory sexual selection occurs as sperm from different males can be stored in the SSTs and compete for fertilization. In this case, it is commonly reported that the last male in the mating sequence has a competitive advantage, as it has a higher likelihood of fertilizing the eggs (Birkhead, 1998a). This widespread phenomenon is known as the last male precedence (LMP) (Birkhead, 1998b).

The mechanisms accounting for the competitive advantage of the last male have been less well studied, although several hypotheses have been put forward (Birkhead and Biggins, 1998). The first hypothesis assumes that stored sperm are progressively lost over time (at a constant or accelerating rate). Therefore, depending on the time interval between matings, and the rate at which sperm is lost, sperm from the last male are likely to outnumber the sperm from the previous males. The second hypothesis assumes that during successive matings, the newly ejaculated sperm will displace the previously stored sperm. Therefore, sperm from the last male merely replace previously stored sperm inside the SSTs. The third hypothesis assumes that sperm are stratified inside the SSTs depending on the mating order. According to this hypothesis, sperm from the first male are stored deep in the SSTs, while sperm of the last male are stored closer to the SST opening and therefore will be released first for fertilization. A final hypothesis assumes that SSTs closer to the vagina are filled with sperm from the first mating male, whereas sperm from the last male are stored in SSTs closer to the uterus (because those close to the vagina are already filled). When this spatial segregation occurs, sperm stored closer to the fertilization site might have a competitive advantage compared with those stored more distally (Briskie, 1996b).

Sperm loss has been considered as the predominant mechanism explaining LMP in birds (Birkhead and Biggins, 1998). However, only a handful of studies have used direct methods to differentiate sperm of different males inside the storage structures, which limits our understanding of LMP in birds. Van Krey et al. (1981) performed inseminations with a combination of radiolabelled (^3^H-thymidine) and unlabelled sperm in the chicken and found no evidence of sperm displacement. King et al. (2002) performed tandem inseminations with sperm labelled with Hoechst 33342 and unstained sperm in chickens and turkeys and showed that sperm from subsequent inseminations segregated in different SSTs. Finally, more recently, Matsuzaki et al. (2023) used a double fluorescent sperm labelling to investigate LMP in chicken. They found that after two consecutive inseminations with a three-day interval, more than 70% of sperm from the first insemination were replaced by sperm of the second one. They also reported evidence showing that the number of stored sperm decreased over the first 5 days after insemination. Based on this sparse experimental evidence, it is difficult to have a clear picture of the relative importance of the mechanisms underlying LMP in birds. Work done on invertebrates provides additional information on the proximal factors underlying LMP. For instance, using transgenic lines of *Drosophila melanogaster* expressing fluorescent proteins on sperm, Manier et al. (2010) showed that sperm of the first male are physically displaced by sperm of the second one, in agreement with the displacement hypothesis.

As mentioned above, testing the different hypotheses underlying the LMP is challenging because we need to differentiate sperm from different males within the SSTs. Fluorescent staining allows to label sperm and visualize them inside SSTs. However, fluorochromes must meet a series for criteria to be suitable for *in vivo* studies. In particular, the staining must label all sperm in the ejaculate, not impair sperm motility or viability and the fluorescent signal should last long enough to allow sperm visualization in the storage structures (Henning et al., 2019).

Here, we aimed to investigate the mechanisms underlying the LMP in a wild bird species with a polyandrous mating system, the North African houbara bustard (*Chlamydotis undulata*). The natural populations of this species have declined over the past decades because of habitat destruction and overhunting and it is classed as vulnerable on the International Union for Conservation of Nature Red List (https://www.iucnredlist.org/species/22728245/208501099). An *ex-situ* conservation breeding program was established in Morocco to restore declining populations through translocation of captive bred houbara (Lacroix, 2003). During the breeding season, lasting from January to June in Morocco, male houbara bustards display in specific sites (exploded leks; Hingrat and Saint Jalme, 2005), and females choose their mate (Hingrat et al., 2007). Females can copulate with several males as multiple paternities have been reported in the wild (Lesobre et al., 2010). Females lay one clutch of 2–4 eggs with a delay of 1 to 3 days between eggs. If a clutch is lost a replacement clutch is laid (Saint Jalme et al., 1994). The reproduction in captivity is artificial with the insemination of females using sperm collected from males housed separately. Using data collected over the past 20 years, Sorci et al. (2023) showed that when females are inseminated with different males, the last male in the insemination sequence has >60% likelihood of fertilizing the eggs, showing that the LMP is operating in this species.

To unravel the mechanisms driving the LMP in the North African houbara bustard and to investigate whether sperm from different males can be found on the egg perivitelline membrane, we first conducted *in vitro* experiments to assess the suitability of different fluorescent dyes. Then, we conducted *in vivo* experiments involving the artificial insemination of females with stained sperm. Specifically, we formulated the following predictions. If passive sperm loss leads to the LMP, the number of sperm stored in the SSTs should decrease with time post-insemination. If sperm displacement occurs, sperm stored in the SSTs from the last insemination should outnumber sperm from the first insemination. If sperm stratify inside SSTs, sperm from the first insemination should be at the bottom of SSTs whereas sperm from the second insemination should be closer to the tubule opening. If sperm segregation occurs, sperm from different inseminations should be separately stored inside different SSTs.

## MATERIALS AND METHODS

### Experimental birds

All North African houbara bustard (*Chlamydotis undulata* Jacquin 1784) used in this study were part of a conservation breeding program at the Emirates Center for Wildlife Propagation (ECWP), in eastern Morocco, aimed at reinforcing natural populations (Lacroix, 2003). All birds were kept in 4 m^2^ individual cages and received commercial specially formulated dry food pellets, mealworms and crickets as food. All males used for the *in vitro* sperm staining experiments and the *in vivo* experiments were part of the breeding flock. The females used for the *in vivo* experiments were born in captivity and were of generation F3 or more. They were due to be culled for the purposes of management of the captive flock; no bird that could have either been released in the wild or kept as a future breeder was euthanized. A total of 22 sexually mature females with a mean age (±s.d.) of 5.45±1.05 years were used in this study.

### Semen collection

Ejaculates were collected with a dummy female presented to the male. When the male mounted the dummy and started copulating, a glass Petri dish was used to collect the ejaculate by pressing it gently on the male cloaca. The ejaculate was immediately transferred to a 2 ml cryotube and diluted (1:1) in Lake 7.1 diluent (Lake and Ravie, 1984). Samples were maintained at room temperature (20°C, <1 h) until they were processed for fluorescent staining.

### *In vitro* experiments

To distinguish sperm of different males, we first evaluated two fluorescent stains: Hoechst 33342 (Invitrogen, USA) and NUCLEAR-ID^®^ Red DNA (Enzo, USA), hereafter called HBlue and NRed, respectively. Given that NRed impaired sperm motility and velocity (see below), we also considered a third stain, MitoTracker™ Red FM (Invitrogen, USA), hereafter called MRed.

#### Effect of HBlue and NRed staining on sperm motility and velocity

**Fig. 1.**
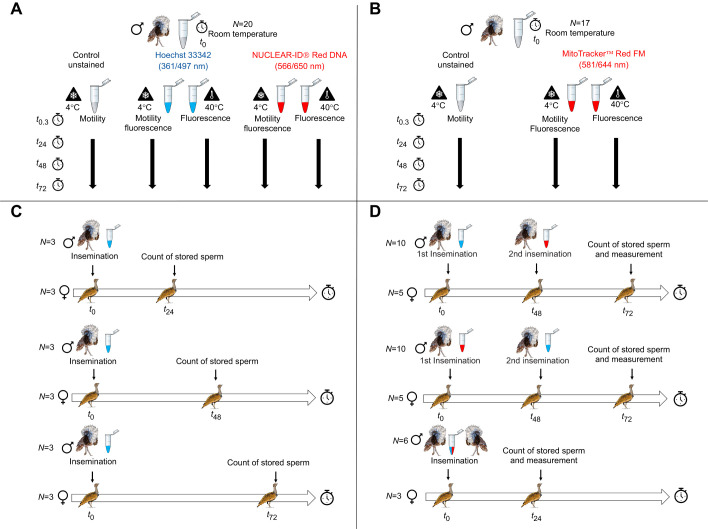
**Experimental designs used for the *in vitro* and *in vivo* experiments, using captive male and female houbara bustards (*Chlamydotis undulata*).** (A) Ejaculates (*n*=20) were split into three aliquots and stained with Hoechst 33342 (HBlue), NUCLEAR-ID^®^ Red DNA (NRed) or left unstained. Sperm motility and velocity were measured at different time points post-staining at 4°C. The persistence of the fluorescent signal was also assessed at 40°C. (B) Ejaculates (*n*=17) were split into two aliquots and stained with MitoTracker™ Red FM (MRed) or left unstained. Sperm motility and velocity were measured at different time points post-staining at 4°C. The persistence of the fluorescent signal was also assessed at 40°C. (C) Nine females were inseminated with HBlue-stained sperm and the number of stained sperm stored in the sperm storage tubules counted at 24, 48 and 72 h post-insemination (*n*=3 females per post-insemination timing). (D) Ten females were inseminated in sequence with either HBlue or MRed stained sperm and after 48 h were inseminated again with MRed sperm (if the first insemination involved HBlue sperm) or with HBlue sperm (if the first insemination involved MRed sperm) (*n*=5 for each group). Twenty-four hours after the last insemination, the number of stained sperm stored in the sperm storage tubules (SSTs) was counted. A control group was constituted of three females that were simultaneously inseminated with a mix of HBlue and MRed stained sperm. Twenty-four hours after the insemination, the number of stained sperm stored in the SSTs was counted.

Ejaculates from 20 males (final concentration=552±110×10^6^ sperm ml^−1^; mean ±s.d.) were used. Each sample was split into three aliquots, one was stained with HBlue (350 nm excitation/461 nm emission), one with NRed (566 nm excitation/650 nm emission) and the third aliquot was used as an unstained control (Fig. 1A). Both fluorochromes had a final concentration of 20 µmol l^−1^. After incubation for 20 min in the dark, at room temperature (20°C), samples were washed (centrifuged for 5 min at 1000 ***g***), resuspended in PBS/1.5% BSA and sperm motility and velocity was assessed. Then, each aliquot was divided in two and kept either at 4°C or 40°C. The persistence of the fluorescence signal was assessed under the microscope 24, 48 and 72 h post-incubation. Motility and velocity were also assessed 24, 48 and 72 h post-incubation but only on samples kept at 4°C. Indeed, although 40°C corresponds to the mean body temperature, at this temperature sperm progressively become immotile (sperm are also immotile inside the SSTs). Avian sperm storage at low temperature is commonly used to preserve motility and limit bacterial growth (Blesbois and Brillard, 2007; Wishart, 2009).

Sperm motility and velocity were assessed with Sperm Class Analyzer software (SCA, Microptics, Barcelona, Spain). One aliquot from each treatment (HBlue, NRed and unstained controls at each time interval) was diluted (1:10 in PBS/1.5% BSA) and 4 μl were placed on a 20 µm depth disposable chamber (Proiser, Arquimea, Spain) under a negative phase contrast microscope (Nikon E200-LED, Japan, objective ×10). A maximum of 30 fields were analysed to reach 500 sperm (see Table S1 for details on SCA settings). We recorded the percentage of motile sperm and their curvilinear velocity [VCL (µm s^−1^)].

#### Effect of MRed staining on sperm motility and velocity

Ejaculates from 17 males (final concentration=278±105×10^6^ sperm ml^−1^; mean ±s.d.) were used. Each sample was split into two aliquots, one was stained with MRed (581 nm excitation/644 nm emission) at a final concentration of 1 µmol l^−1^ and the other aliquot was used as an unstained control (Fig. 1B). After incubation for 20 min in the dark at room temperature (20°C), samples were washed (centrifuged for 5 min at 1000 ***g***), resuspended in PBS/1.5% BSA and sperm motility and velocity were assessed as described above.

### *In vivo* experiments

#### Sperm loss

To investigate sperm loss over time, nine females were inseminated with 40×10^6^ sperm stained with HBlue. Females were euthanized at 24, 48 and 72 h post-insemination (*n*=3 females per post-insemination time) to count the number of stained sperm stored in the SSTs (Fig. 1C). Birds were euthanized by veterinarians of the ECWP by an intravenous injection of 1 ml of T61 (MSD, 200 mg Embutramide, 50 mg mebezonium iodide and 5 mg tetracaine hydrochloride) (Baker-Cook et al., 2021). Immediately after euthanasia, the coelom was opened, the oviduct harvested, and the utero vaginal junction (UVJ) was removed, placed in 1×PBS and transferred to the laboratory. The UVJs were dissected under a stereomicroscope (MZ9.5, Leica, Switzerland) within 1 h of collection. Mucosa folds containing SSTs were identified with Leica Application Suite software EZ software (version 3.2.1, Leica, Switzerland). Mucosa folds were placed on poly-L-lysine coated slides with one drop of mounting medium (1:1 Glycerol and 1×PBS) and a cover slip. Slides were observed under a fluorescent microscope (Nikon E200-LED, Japan; fluorescent light: CoolLED pE-300-W-EPI-Fluorescence LED illumination system and module: Nikon EPI-Fluorescence module CI-FL) equipped with a camera. For each female, a maximum of 100 SSTs (containing at least one sperm) were observed and photographed using a blue filter (Chroma AT-UV/DAPI Longpass filter block) and a red filter (Texas Red filter). The number of stained sperm in each SST was then counted on the photographs.

#### Sperm displacement, segregation, and stratification

To investigate sperm displacement, stratification and segregation, 10 females were either first inseminated with 40×10^6^ HBlue-labelled sperm, and after 48 h, with 40×10^6^ MRed-labelled sperm, or first inseminated with 40×10^6^ MRed sperm and after 48 h with 40×10^6^ HBlue sperm (five females per group) (Fig. 1D). Twenty-four hours after the last insemination, females were euthanized, and the oviduct collected and processed as described above. The number of HBlue sperm and the number of MRed sperm was counted in each SST.

To control for a possible negative effect of the staining on sperm attributes, a control group was formed by three females that were simultaneously inseminated with a mix of 20×10^6^ HBlue and 20×10^6^ MRed sperm collected from two males. These females were also euthanized 24 h post-insemination and processed as described above.

To assess the position of stained sperm within SSTs, a total of ten SSTs were selected for each female and the distance (µm) of each sperm (head and midpiece junction used as measuring point) from a fixed point of the dead end of the SST was computed using Fiji software (Schindelin et al., 2012, December 2020 version). The distance was measured using segmented lines to adjust to the shape of the SSTs.

#### Stained sperm on the perivitelline membrane

One female was inseminated with a mix of 20×10^6^ HBlue- and 20×10^6^ MRed-stained sperm collected from two males and was allowed to lay. One egg was collected the day of laying and processed as to visualize the presence of stained sperm. Another egg was found inside the uterus of one of the control females inseminated with a mix of stained sperm and used to visualize the presence of stained sperm.

Eggs were assessed for fertility and the presence of sperm on the perivitelline layer. Briefly, the yolk was transferred inside a glass container filled with 1×PBS. The perivitelline membrane surrounding the embryonic disk was cut with fine scissors and after removing yolk excess, was placed on poly-L-lysine coated slides with one drop of mounting medium (1:1 Glycerol and 1×PBS) and a coverslip. The presence of stained sperm was checked under fluorescent microscope (Nikon E200-LED, Japan; fluorescent light: CoolLED pE-300-W-EPI-Fluorescence LED illumination system and module: Nikon EPI-Fluorescence module CI-FL) using a blue (Chroma AT-UV/DAPI Longpass filter block) and a red (Texas Red filter) filter. The presence of embryonic cells was later checked by adding a drop of DAPI mounting medium (Fluoroshield, Abcam, USA) on the slide.

#### Statistical analyses

For the *in vitro* experiments, we investigated the effect of the staining treatment on sperm motility and velocity (VCL) using general linear mixed models with a normal distribution of errors. We first ran models on sperm motility and VCL measured after the 20 min incubation with the stains. These models included the staining treatment (unstained, HBlue, NRed; unstained, MRed) as a fixed effect and the ejaculate identity as a random effect. The random effect was included because the same ejaculate was used for the different staining treatments. We then ran models on sperm motility and VCL was measured at different time points post-staining. These models included the staining treatment (unstained, HBlue, NRed; unstained, MRed), time post-staining (24, 48, 72 h, considered as a factor), and the two-way interaction between staining treatment and time post-staining as fixed effects. The models also included the ejaculate identity as a random effect for the reason mentioned above.

For the *in vivo* experiments, we first investigated whether the number of sperm counted in the SSTs decreased over time post-insemination. This was done by running a generalized linear mixed model with a negative binomial distribution of errors. The response variable was the count of sperm per SST. Time post-insemination (24, 48, 72 h, considered as a factor) was included as a fixed effect, and the female identity was included as a random effect. Female identity was included as a random effect because multiple SSTs were screened per female.

To investigate whether, following sequential inseminations, sperm inseminated first or inseminated last were differentially stored in SSTs, we ran a generalized linear model with a negative binomial distribution of errors. The response variable was the count of sperm per SST. The insemination order (first or last), the staining treatment (HBlue or MRed), and the two-way interaction were included as fixed effects. The model also included two random effects: the female identity and the SST identity nested within the female identity. This was done because HBlue and MRed sperm were counted in each SST (therefore each SST had two sperm counts), and several SSTs were screened per female. To investigate whether, following a single insemination with a mix of HBlue and MRed sperm, the staining treatment affected the number of sperm stored in the SSTs, we ran a generalized linear mixed model with a negative binomial distribution of errors. The response variable was the count of sperm per SST, the staining treatment was included as a fixed effect. The model also included female identity and SST identity nested within female identity for the reason explained above.

To investigate whether sperm from the first or the last insemination segregated in different SSTs, we computed the proportion (p1) of SSTs with sperm from both inseminations, the proportion (p2) of SSTs with sperm from the first insemination alone and the proportion (p3) of SSTs with sperm from the last insemination alone, over the whole sample of 10 females. These proportions were then compared to the null hypothesis of equal proportions (p1=p2=p3=0.33). This was done using an overall goodness of fit test, but also using a Dirichlet-multinomial model that allows to account for between-female variability (as mentioned above, several SSTs were screened per female). We also ran these two tests (the overall goodness of fit test and the Dirichlet-multinomial model) on the restricted sample of SSTs that only contained either sperm from the first insemination, or sperm from the last insemination. In this case, we compared the observed proportions of tubules containing HBlue sperm from the first insemination (p1), HBlue sperm from the last insemination (p2), MRed sperm from the first insemination (p3), MRed sperm from the last insemination (p4) to the null hypothesis of equal proportions (p1=p2=p3=p4=0.25).

To investigate whether, following sequential inseminations, sperm from the first or the last insemination were located at different distances from the bottom of the SSTs, we ran a general linear mixed model with a normal distribution of error. The response variable was the distance (µm) of each sperm from the bottom of the tubule. The staining treatment (HBlue or MRed), the insemination order (first or last) and the two-way interaction were included as fixed effects. The model also included female identity and SST identity nested within female identity because the distance from the bottom of several sperm was measured for each SST and several SSTs were screened per female. In the case of inseminations with mixes of HBlue and MRed stained sperm, the sperm distance from the bottom of the SST was analysed as described above with the difference that the model only included the staining treatment as a fixed effect.

All statistical analyses were done in SAS studio.

#### Ethical statement

The breeding program runs under the approval of the Moroccan Ministry of Agriculture and a mandate independent veterinarian (mandate number: 534-98), providing control of the well-being of the birds. Onsite veterinary facilities ensure the best possible care for sick or injured birds by a team of expert veterinarians, and standards from sanitary authorities are regularly controlled. Experiments were conducted in an ethical manner, according to the Directive 2010/63/EU of the European Parliament and of the Council on the protection of animals used for scientific purposes (http://data.europa.eu/eli/dir/2010/63/oj), and each breeding protocol is developed while ensuring appropriate and ethical treatment of birds. To minimize stress during capture, birds are fitted with hoods during manipulation. Facilities are designed to ensure the well-being of the birds and prevent injuries.

## RESULTS

### *In vitro* experiments

#### Effect of HBlue and NRed staining on sperm motility and velocity

**Fig. 2.**
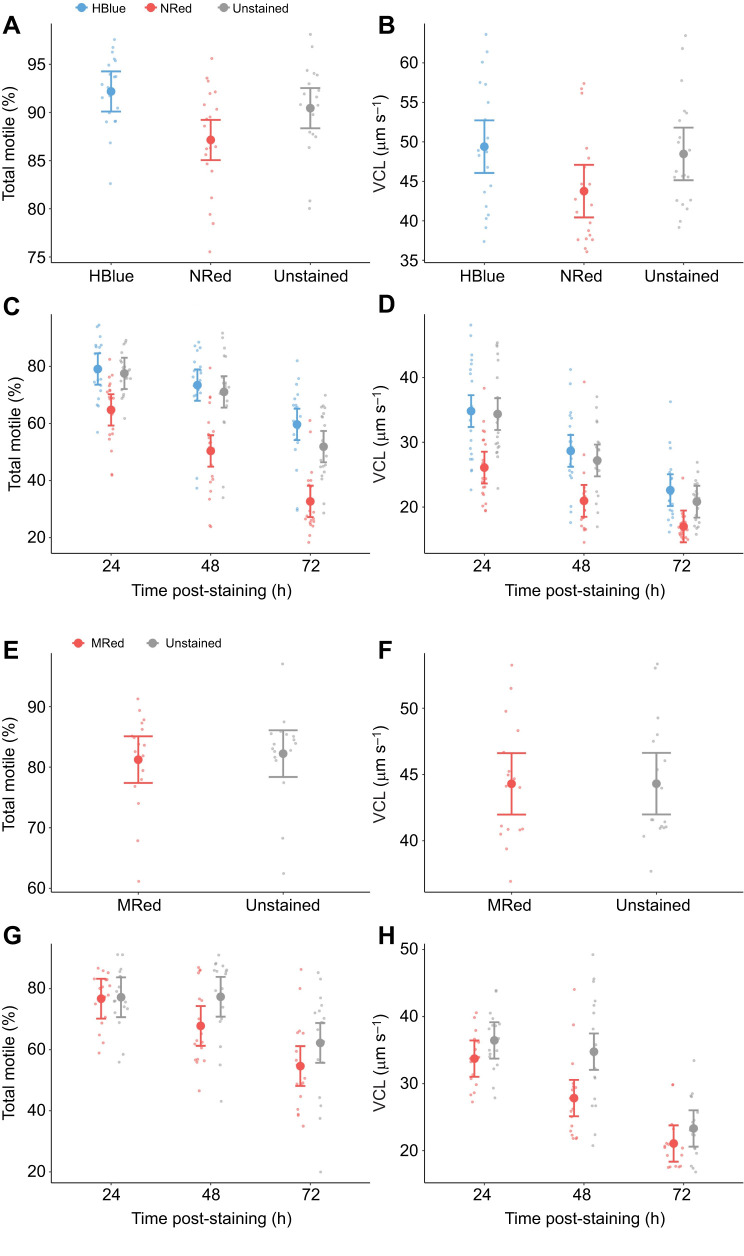
**Total motility and curvilinear velocity of stained sperm in the houbara bustard.** (A–D) Total motility (%) (A,C) and curvilinear velocity (VCL in µm s^−1^) (B,D) after 20 min incubation at room temperature in the dark (A,B) or 24, 48 and 72 h of incubation at 4°C in the dark (C,D) of sperm stained with 20 µmol l^−1^ HBlue, 20 µmol l^−1^ NRed or unstained. Values are expressed as raw data (smaller dots), marginal means (larger dots) and lower and upper 95% confidence intervals (lower and upper error bars) (*n*=20 ejaculates). (E–H) Total motility (E,G) and VCL (F,H) after 20 min incubation at room temperature in the dark (E,F) or after 24, 48 and 72 h of incubation at 4°C in the dark (G,H) of sperm stained with 1 µmol l^−1^ MRed or unstained. Values are expressed as raw data (smaller dots), marginal means (larger dots) and lower and upper 95% confidence intervals (lower and upper error bars) (*n*=17 ejaculates).

After incubation at room temperature for 20 min, there were no statistically significant differences in total motility (Fig. 2A) and VCL (Fig. 2B) between sperm stained with HBlue and unstained sperm (Table S2). However, sperm stained with NRed had statistically significant lower motility and VCL compared with unstained sperm (Table S1). Over time, motility decreased at a statistically significant faster rate for sperm stained with NRed than for sperm stained with HBlue or unstained sperm (Table S3; Fig. 2C). The rate of VCL decline over time was not statistically different between the three staining treatments (Table S3; Fig. 2D). The fluorescent signal persisted for up to 72 h at both temperatures (4 and 40°C) for the two fluorochromes (Fig. S1).

#### Effect of MRed staining on sperm motility and velocity

After incubation for 20 min at room temperature, there were no statistically significant differences in total motility (Fig. 2E) or VCL (Fig. 2F) between sperm stained with MRed and unstained sperm (Table S4). Total motility (Fig. 2G) and VCL (Fig. 2H) decreased with time at a statistically significant faster rate for sperm stained with MRed than for unstained sperm (Table S5). The fluorescent signal persisted for up to 72 h at both temperatures (4 and 40°C).

### *In vivo* experiments

#### Sperm loss

**Fig. 3.**
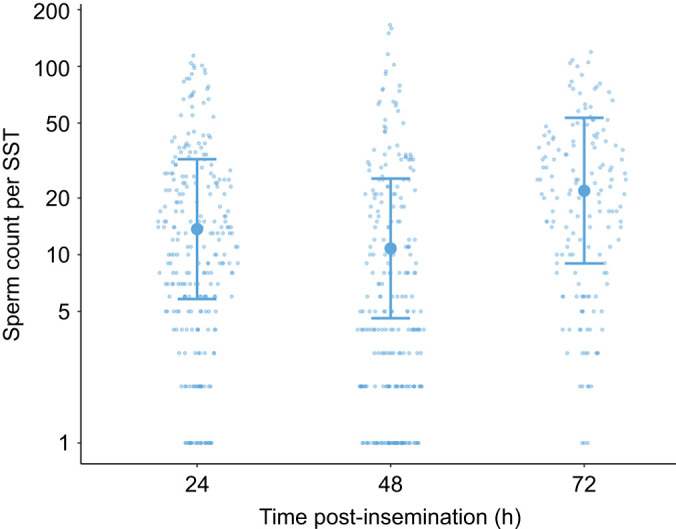
**Number of houbara bustard sperm counted per sperm storage tubule (SST) at 24, 48 and 72 h post-insemination.** Values are plotted on a log_10_ scale and expressed as raw data (smaller dots), marginal means (larger dots) and lower and upper 95% confidence intervals (lower and upper error bars) (*n*=3 females and *n*=253 SSTs at 24 h, *n*=3 females and *n*=248 SSTs at 48 h, *n*=3 females and *n*=156 SSTs at 72 h).

The number of stained sperm counted in the SSTs did not significantly change over time, up to 72 h post-insemination (Table S6; Fig. 3).

#### Sperm displacement, segregation and stratification

Sperm stained with HBlue appeared with a bright blue nucleus and sperm stained with MRed had a red midpiece. Sperm with a red midpiece also showed a light blue nuclear staining due to HBlue stain transfer. However, this did not prevent us from assigning sperm to the two stains. HBlue and MRed sperm were found together in 85% of the SSTs of females inseminated with sperm mixes. Significantly more sperm stained with HBlue were found in the SSTs after insemination with sperm mixes (Table S7; Fig. S2A,C).

**Fig. 4.**
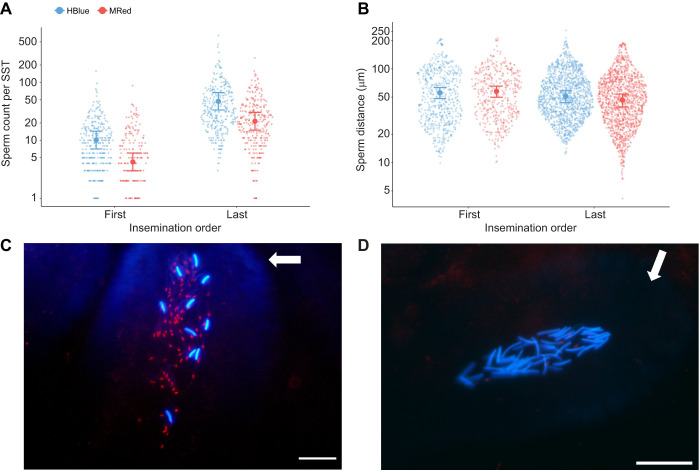
**Number of sperm counted per SST according to the insemination order and the staining treatment.** (A) Values are plotted on a log_10_ scale and expressed as raw data (smaller dots), marginal means (larger dots) and lower and upper 95% confidence intervals (lower and upper error bars) (*n*=5 females and 317 SSTs for HBlue first/MRed last, *n*=5 and 300 SSTs for MRed first/HBlue last). (B) Sperm distance (µm) from the bottom of SSTs according to the insemination order and the staining treatment. Values are plotted on a log_10_ scale and expressed as raw data (smaller dots), marginal means (larger dots) and lower and upper 95% confidence intervals (lower and upper error bars) (HBlue first: *n*=5 females and *n*=50 SSTs; *n*_HBlue_ =610 and *n*_MRed_=416; MRed first: *n*=5 females and *n*=46 SSTs; *n*_HBlue_=1400 and *n*_MRed_=451). (C,D) Photomicrographs of SSTs of a female first inseminated with HBlue and then with MRed (C) stained sperm and a female first inseminated with MRed and then with HBlue stained sperm (D) (48 h interval between inseminations) illustrating the higher number of sperm from the second insemination. Magnification ×400, merged blue and red fluorescence, and white arrow indicates the dead end of the SST. Scale bars: 20 µm.

Females inseminated at 48 h intervals stored significantly more sperm stained with HBlue (Table S8). However, females consistently stored more sperm from the last insemination, independently of the staining treatment (Table S8), and the interaction between insemination order and staining treatment was not statistically significant (Table S8; Fig. 4A,C,D).

We did not find evidence suggesting that sperm from first and last inseminations might segregate in different SSTs. Under the hypothesis of spatial segregation, we would expect a majority of SSTs containing either sperm from the first or sperm from the last insemination. Over the whole sample of SSTs screened in the 10 females, we found that 79.1% (488/617) contained sperm from both inseminations, 3.08% (19/617) contained sperm from the first insemination alone, and 17.83% (110/617) contained sperm from the last insemination alone. These proportions are significantly different from a random one-third expectation, both when using an overall goodness of fit test (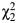
=601.56, *P*<0.01), or when using a Dirichlet–multinomial model accounting for between-female variability (likelihood ratio test, LRT=20.3, d.f.=2, *P*<0.01). Among the SSTs that only had sperm from one of the two inseminations, there was a clear skew towards SSTs containing HBlue-stained sperm from the last insemination [HBlue last=80.62% (104/129), HBlue first=14.73% (19/129), MRed last=4.65% (6/129), MRed first=0% (0/129)] (overall goodness of fit test, 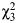
=218.69, *P*<0.01; Dirichlet–multinomial model accounting for between-female variability, LRT=9.2, d.f.=3, *P*=0.03).

According to the stratification hypothesis, sperm should be chronologically stored in the SST from the bottom (first insemination) to the opening (last insemination). To explore this hypothesis, we measured the distance of each sperm from the bottom of the SST. The stratification hypothesis predicts that this distance should be smaller for first inseminated sperm. Contrary to this prediction, we found that sperm from the second insemination were significantly closer to the bottom of the SST compared with sperm from the first insemination (Table S9, Fig. 4B). Staining treatment did not affect the position of sperm within the SSTs (Table S9, Fig. 4B) and the interaction between insemination order and staining treatment was not statistically significant (Table S9). In mixed inseminations, the distance from the bottom of the SST did not significantly differ between sperm stained with HBlue and MRed (Table S10, Fig. S2B).

#### Stained sperm on the perivitelline membrane

The egg laid by the female inseminated with a mix of HBlue and MRed stained sperm had been fertilized since embryonic cells were observed upon adding DAPI on the slide. We also found several sperm with a light blue nucleus and a red midpiece on the egg membrane, corresponding to the MRed-stained sperm. An additional egg was found inside the uterus of one female inseminated with a mix of HBlue- and MRed-stained sperm. Both MRed- and HBlue-stained sperm were found on the egg membrane (Fig. S3). As for the previous egg, embryonic cells were also observed in this egg, showing that it had been successfully fertilized.

## DISCUSSION

Although the LMP has been reported in a diversity of organisms, the mechanisms underlying it have been difficult to elucidate. Several hypotheses have been put forward, including the passive loss of sperm stored in the SSTs and the active displacement of stored sperm during successive matings. However, testing these hypotheses has proved challenging because of the technical difficulties in visualizing competing sperm within the female reproductive tract. After conducting *in vitro* experiments to assess the suitability of different fluorescent stains, we used artificial inseminations with fluorescent stained sperm in the North African houbara bustard and showed that the displacement of sperm stored in the SSTs probably accounts for the last male precedence in this species.

To effectively stain sperm, we tested two nuclear fluorescent dyes and one mitochondrial stain. Total motility and VCL of sperm stained with HBlue were not statistically significantly different from unstained sperm over time. Moreover, HBlue produced a fluorescent signal that persisted for up to 72 h. Therefore, this fluorochrome was considered as a good candidate for *in vivo* use. This staining has also been used in the past in studies conducted on chickens (McDaniel et al., 1997; King et al., 2002; Matsuzaki et al., 2023), turkeys (Bakst, 1994) and Japanese quail (Matsuzaki et al., 2021) and no sperm motility impairment has been reported. It is also commonly used to stain mammalian sperm without impacting motility and it is even used in assisted reproduction techniques for sperm sex-sorting (Garner et al., 2013). The second nuclear staining that we tested, NRed, did impair motility and VCL and therefore was not considered suitable for *in vivo* use. In a recent paper, Matsuzaki et al. (2023) used two stains, a nuclear one (HBlue) and a cytosolic one (pHrodo-Red AM) to conduct artificial inseminations. However, pHrodo-Red AM emits a red fluorescence that depends on the pH of the cytosol, the stronger the acidity the higher the intensity of the signal. Since we do not have data on the pH of the oviduct in the houbara bustard, we preferred to use dyes emitting fluorescence independently of pH conditions. Thus, we tested a mitochondrial staining (MRed) and found that motility and VCL were similar, 20 min after staining, for stained and unstained sperm. However, both motility and velocity decreased at a faster rate (from 24 to 72 h post-insemination) compared with unstained sperm. Once sperm are stored inside the SSTs they enter into a resting phase and lose motility until fertilization of the oocyte (Matsuzaki and Sasanami, 2022). Therefore, despite the faster rate of decrease in motility and VCL of MRed-stained sperm, we chose the mitochondrial staining for *in vivo* use, based on the rationale that 24 h post-staining sperm will be already stored in the SSTs (where they are no longer motile). The validity of the choice of the two fluorochromes for the *in vivo* experiments was confirmed by the observation of both MRed- and HBlue-stained sperm, not only inside the SSTs, but also on the perivitelline membrane. Up to now, only three fluorescent molecules, Hoechst 33342 (HBlue) (King et al., 2002; Matsuzaki et al., 2021, 2023), pHrodo-Red AM (Matsuzaki et al., 2021, 2023) and MRed (present study) have been successfully used to study sperm storage in a competitive insemination context.

The results of the *in vivo* experiments suggest that there is no sperm loss over time, up to 72 h post-insemination. Therefore, for successive matings occurring within a period of 2–3 days, passive sperm loss is unlikely to account for the LMP effect. Passive sperm loss certainly operates on a longer time scale given that, although females can lay fertile eggs up to 47 days post-insemination, fertility decreases over time after insemination (Saint Jalme et al., 1994). Previous work has suggested that passive sperm loss might be the main mechanism underlying LMP in birds (Birkhead and Biggins, 1998). To reach this conclusion, Birkhead and Biggins (1998) built a series of models that included passive sperm loss alone and passive sperm loss plus sperm stratification or displacement and used them to predict the expected paternity share under the different scenarios modelled. They then compared these predicted paternity shares with the observed ones derived from four published studies on poultry. The model that included the passive sperm loss alone provided the best fit to the observed paternity share (Birkhead and Biggins, 1998). However, it should be noted that the model that included passive sperm loss plus displacement provided relatively similar predictions to the model with passive sperm loss alone and it might be very difficult to tease apart the two models using datasets that have not been collected with the specific aim of testing them. Therefore, a direct assessment of the rate of sperm loss in the SSTs is needed to conclude on the importance of sperm loss compared with the other mechanisms.

When inseminated with a mix of sperm stained with the two fluorochromes, we expected to retrieve the same number of sperm in the SSTs. Contrary to this prediction, we found more sperm stained with HBlue compared with sperm stained with MRed. This difference is probably due to the effect of MRed on sperm motility, as reported *in vitro*. During the sequential inseminations, the difference in the number of HBlue- and MRed-stained sperm was found again (more HBlue sperm); however, more sperm from the last insemination were consistently found in the SSTs, independently of the staining used (and the interaction between staining and insemination order was not significant). These results, therefore, suggest that sperm transferred during successive matings may replace those previously stored in the SSTs. Therefore, sperm replacement might be at play as a mechanism underlying LMP in the North African houbara bustard. A recent study in the chicken showed that >70% of sperm of a first insemination were replaced by sperm of a second insemination occurring 3 days later (Matsuzaki et al., 2023).

We did not find evidence supporting the spatial segregation hypothesis since the majority of SSTs contained sperm from both the first and the last insemination. This result somehow contradicts previous work on chickens and turkeys (King et al., 2002), which reported that only 4% and 12% of the SSTs contained sperm from different males. We did not find evidence for the stratification hypothesis either. The stratification hypothesis posits that sperm accumulate within the SST, with first transferred sperm being stored at the bottom of the SST and last transferred sperm being stored close to the SST opening. However, we found that sperm of both inseminations were rather thoroughly mixed in the SSTs, and contrary to the prediction, sperm transferred during the last insemination were significantly closer to the bottom of the SSTs compared with sperm transferred during the first insemination. Therefore, this finding does not support the stratification hypothesis.

We also report evidence showing that sperm stained with both fluorochromes were potentially able to fertilize the eggs. Several sperm (stained with the two dyes) were found on the perivitelline membrane and eggs were fertile, as embryonic cells were observed. Polyspermy refers to the entrance of multiple sperm inside the egg; however, only one sperm can fuse with the oocyte nucleus to form a zygote. Polyspermy represents a crucial physiological process in avian reproduction, essential for successful fertilization (Hemmings and Birkhead, 2015). The presence of multiple sperm inside the perivitelline membrane of the egg has been reported in several studies (Wishart, 1987; Brillard and Bakst, 1990; Brillard and Antoine, 1990; Hemmings and Birkhead, 2017). In the turkey, the number of sperm found on the perivitelline membrane appears to be positively correlated with the amount stored inside the SSTs (Brillard and Bakst, 1990). In the zebra finch (*Taeniopygia guttata*), sperm of two males, with different phenotypes, have been found on the perivitelline membrane, the higher the number of sperm of a given phenotype stored in the SSTs, the higher the number found on the PVM (Hemmings and Birkhead, 2017). Here, the number of sperm on the PVM was not quantified, but it would be interesting to investigate whether the number of sperm reaching the egg differs when inseminations occur sequentially. This would provide strong evidence that the competitive advantage of the last male in the mating sequence is due to a larger number of sperm reaching the egg.

This study enabled us to test different LMP mechanisms and provided some evidence in favour of sperm displacement in houbara bustards. However, we would like to point out some limitations of our experimental design. First, the use of the MRed stain to study sperm storage *in vivo* is not ideal, as we found that it had an impact on sperm motility *in vitro*. As mentioned above, HBlue and MRed are currently the only stains that allow sperm to be tracked *in vivo* in houbara, and several options of live cell staining were tested on a pilot phase (data not shown). However, despite the impact of MRed, we found that more sperm from the last inseminations were stored independently of the stain used, confirming the ability of both MRed and HBlue stained sperm to reach SSTs and compete for egg fertilization.

Second, we were unable to test the effect of stains on motility *in vitro* at 40°C, although this corresponds to the mean body temperature sperm experience once they are transferred into the female reproductive tract. Sperm cells become immotile after collection if incubated at 40°C, whether stained or not, and this phenomenon has been reported in several bird species (Ashizawa et al., 2000; Holm and Wishart, 1998).

Third, our results allowed us to discard some of the mechanisms underlying the LMP in the houbara bustard. For instance, we can reject the hypothesis that better fertilization success of the last male in the mating sequence arises owing to loss of sperm from previous matings since we found that the number of sperm stored in the SSTs did not significantly change up to 72 h post-insemination. Based on our results, the most likely mechanism underlying the LMP in the houbara is the displacement of sperm previously stored in the SSTs. However, we should fully acknowledge that our experimental design did not allow us to directly observe sperm from the last insemination actively displacing previously stored sperm. The evidence we provide refers to the fact that more sperm from the last insemination were consistently observed in the SSTs. This finding, combined with the result of no sperm loss over the 72 h post-insemination, is consistent with the sperm displacement hypothesis. That said, we acknowledge that this is indirect evidence in favor of the sperm displacement hypothesis. Moreover, although we do not know how many sperm can be stored in the SSTs, it seems clear that most of them were not completely filled, leaving room for sperm from different inseminations to be stored.

Finally, we acknowledge that, beyond statistical significance, some of the effect sizes were rather small (e.g. the difference in sperm distance from the bottom of the SSTs between first and last inseminations). We therefore invite the reader to cautiously interpret differences based on weak effect sizes.

### Conclusions

Our results are consistent with the hypothesis of sperm displacement as a possible mechanism underlying the competitive advantage experienced by houbara males that mate last in the mating sequence. We did not find evidence supporting the stratification or the segregation hypotheses. Passive sperm loss certainly occurs within the SSTs, as fertilization success declines as long as the delay between insemination and laying increases, with no fertile eggs being laid 47 days post-insemination. This might result from different processes such as the sperm leaking from the tubules or the death and removal of sperm by macrophages. However, we did not find evidence that sperm loss occurs over a short period of time, as the number of stained sperm retrieved at 24, 48 and 72 h post-insemination did not differ significantly. Therefore, for matings occurring within a period of 2–3 days, passive sperm loss is unlikely to account for the LMP effect. We also reported evidence showing that sperm from different males do reach the perivitelline membrane. While polyspermy is a physiological process in birds, the presence of sperm of different males paves the way for competition occurring not only to reach the egg, but also within the egg. Future work should attempt to go further in elucidating the sperm displacement hypothesis, ideally by testing if sperm from previous inseminations are indeed actively displaced by sperm from subsequent inseminations.

## Supplementary Material

10.1242/jexbio.251565_sup1 Supplementary information
