# Supplementary material for: Exploring the mechanisms underlying the last male precedence in the North African houbara bustard
Source: J Exp Biol. 2025 Dec 19;228(24):jeb251565. doi: 10.1242/jeb.251565 (PMC12752492; doi:10.1242/jeb.251565)
Supplement: Supplementary information [file jexbio-228-251565-s1.pdf]

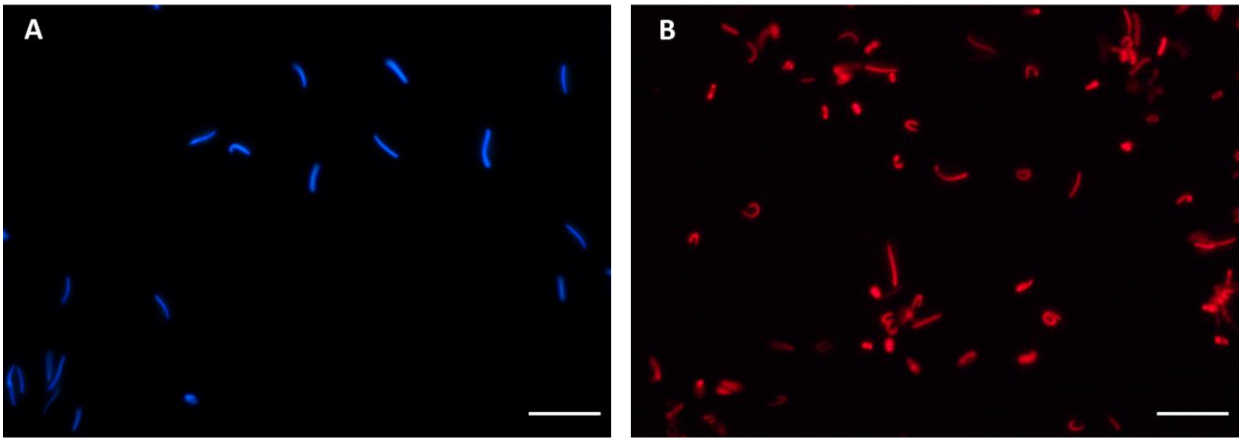

Fig. S1. Photomicrographs of North African houbara bustard (*Chlamydotis undulata*) sperm stained with HBlue (**A**) and with NRed (**B**) at 20 µM concentration after 72 hours of incubation at 40°C. Magnification ×400, observed with HBlue and red fluorescence filters, scale bars represents 20µm.

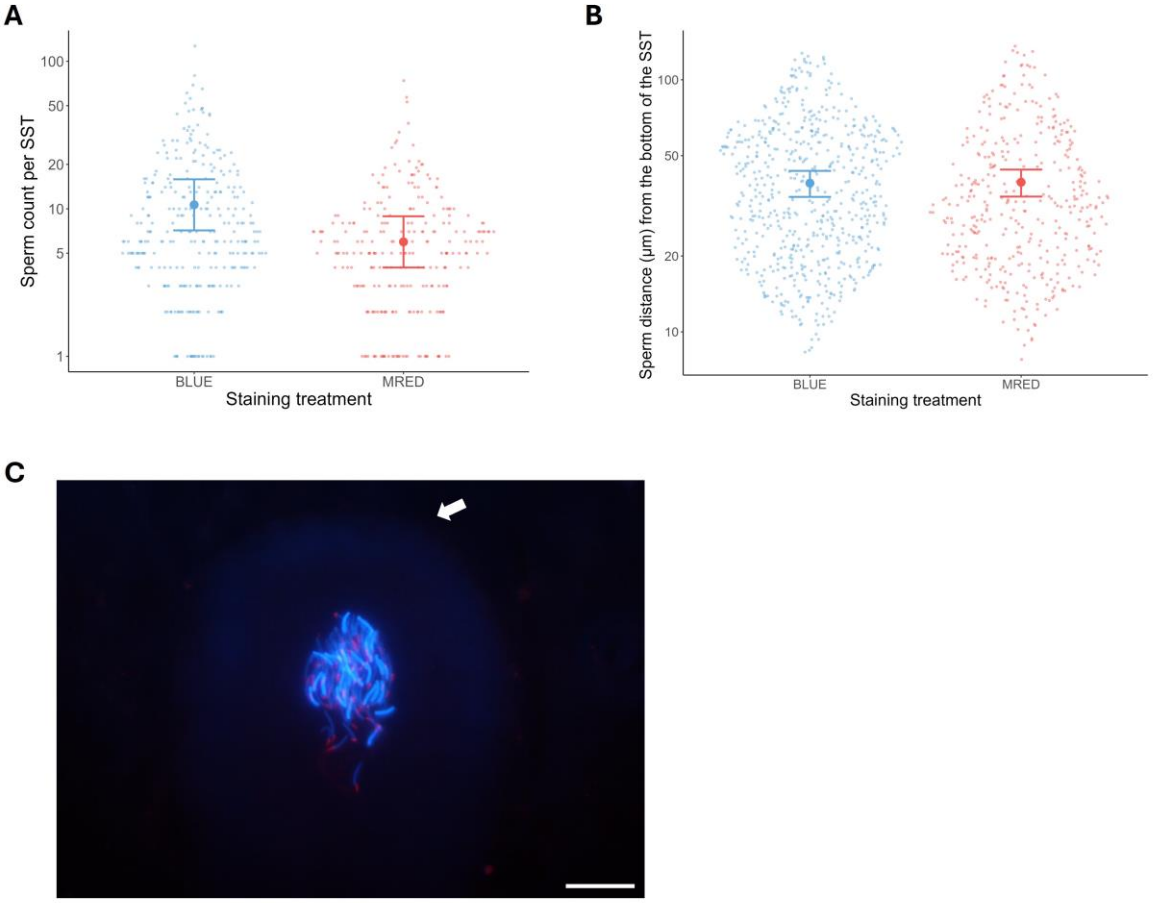

Fig. S2. Number of sperm counted per SST in females inseminated with mixes of HBlue and MRed stained sperm (**A**). Values are plotted on a log10 scale and expressed as raw data (smaller dots), marginal means (larger dots) and lower and upper 95% confidence intervals (lower and upper error bars) (n = 3 females and 300 SSTs). (**B**) Sperm distance (µm) from the bottom of the SSTs in females inseminated with a mix of HBlue and MRed sperm. Values are plotted on a log10 scale and expressed as raw data (smaller dots), marginal means (larger dots) and lower and upper 95% confidence intervals (lower and upper error bars) (n = 3 females, 30 SSTs, n_{HBlue_sperm} = 597, and n_{MRed_sperm} = 383). (**C**) Photomicrograph of a SST of Houbara Bustard (*Chlamydotis undulata*) female containing MRED and HBLUE stained sperm following the insemination with a mix of sperm. Sperm stained with HBLUE have a bright HBlue nucleus while sperm stained with MRed have a bright red midpiece and a faded HBlue nucleus; Magnification ×400, merged HBlue and red fluorescence, scale bar represents 20 µm and white arrow indicates the dead end of the SST.

Journal of Experimental Biology • Supplementary information

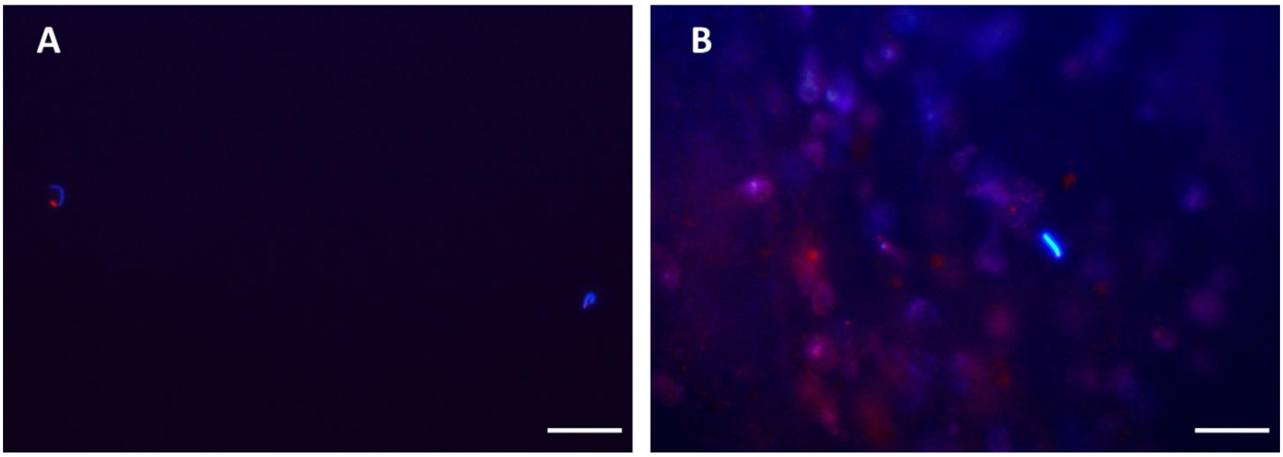

Fig. S3. Photomicrograph of HBlue and MRed stained sperm (**A**) and embryonic cells (**B**) on the perivitelline membrane of an egg found inside the uterus of a North African Houbara Bustard (*Chlamydotis undulata*) female inseminated with a mix of sperm; Magnification ×400, merged HBlue and red fluorescence, scale bars represent 20µm.

Table S1. SCA settings used for the analysis of sperm motility and velocity

Microscope		Capture	
Calibration name	10x	Analysis timeout	15
Calibration value (µm/pixel)	0,420918	Box size (pixels)	152
Capture method	Ph-	Check image	yes
Grid distance (µm)	100	Frame rate (fps)	50
**Disposable**		Number of images	25
Disposable	Proiser	Preview	one second
Depth (µm)	20	Resolution	Low
SS correction factor	1.3	Safe Mode	yes
		Style	Automatic
**Parameters**		**Finish**	
Area (µm2)(min)	5	Sperm to analyze	500
Area (µm2)(max)	190	Fields to analyze	30
Drifting (µm/s)	0	Minimum sperm to analyze	200
Static (µm/s) <	10	Minimum fields to analyze	3
Slow-Medium (µm/s)	50	Warning (%)	20
Rapid (µm/s) >	100		
Progressive (STR>)	70		
Connectivity (pixels)	18		
VAP points (pixels)	5		
VCL/VAP	VCL		

Journal of Experimental Biology • Supplementary information

Table S2. Effect of HBlue and NRed staining on *in vitro* sperm motility and VCL after 20 minutes of incubation. We report the output of general linear mixed models with a normal distribution of errors where the staining treatment was included as a fixed effect and the ejaculate ID as a random intercept (because sperm from the same ejaculate were used for the three staining treatments). For the fixed effect, we report the degrees of freedom, the F and p values. For the random effect, we report the parameter estimate with the standard error, the z and p values. We also report marginal means with the 95% confidence intervals, and the difference of marginal means between staining treatments with the adjusted 95% confidence intervals, t values and Bonferroni adjusted p values. All values were rounded to the second decimal place at the request of an anonymous referee. N = 20 ejaculates and 60 observations for each model.

Total motility (%)

**Fixed effect**				**df**	**F**	**p**
Staining treatment				2,38	11.17	<0.01
**Random effect**		**Estimate**	**SE**	**z**	**p**	
Ejaculate ID		9.61	4.48	2.15	0.02	
**Marginal means**		**Mean**	**95% CI**			
	unstained	90.45	88.36/92.54			
	HBlue	92.19	90.09/94.28			
	NRed	87.15	85.05/89.24			
**Difference of marginal means**		**Estimate**	**95% CI**	**t**	**p**	
Staining treatment						
	HBlue - unstained	1.74	-0.98/4.45	1.60	0.35	
	NRed - unstained	-3.30	-6.02/-0.59	-3.05	0.01	
	HBlue - NRed	5.04	2.33/7.75	4.65	<0.01	

VCL (μm/s)

**Fixed effect**				**df**	**F**	**p**
Staining treatment				2,38	21.28	<0.01
**Random effect**		**Estimate**	**SE**	**z**	**p**	
Ejaculate ID		43.40	15.03	2.89	<0.01	
**Marginal means**		**Mean**	**95% CI**			
	unstained	48.47	45.20/51.73			
	HBlue	49.39	46.13/52.66			
	NRed	43.76	40.49/47.02			
**Difference of marginal means**		**Estimate**	**95% CI**	**t**	**p**	
Staining treatment						
	HBlue - unstained	0.92	-1.40/3.24	1.00	0.97	
	NRed - unstained	-4.71	-7.03/-2.39	-5.09	<0.01	
	HBlue - NRed	5.64	3.76/7.51	6.08	<0.01	

Table S3. Effect of HBLUE and NRED staining on *in vitro* sperm motility and VCL over time, up to 72 hours post-staining. We report the output of general linear mixed models with a normal distribution of errors where the staining treatment, the time post-staining (hours) and the two-way interaction were included as fixed effects and the ejaculate ID as a random intercept (because sperm from the same ejaculate were used for the three staining treatments). For the fixed effects, we report the parameter estimates with the 95% confidence intervals, the degrees of freedom, the F and p values. For the random effect, we report the estimate with the standard error, the z and p values. For total motility, we also report the marginal means with the 95% confidence intervals for the combination of staining treatment x timing post-insemination since the two-way interaction was statistically significant. For VCL, we report the marginal means with the 95% confidence intervals for the staining treatment and timing post-insemination separately since the interaction was not statistically significant. All values were rounded to the second decimal place at the request of an anonymous referee. N = 20 ejaculates and 180 observations for each model.

Total motility (%)

**Fixed effects**			**Estimate**	**95% CI**	**df**	**F**	**p**
Staining treatment					2,152	96.59	<0.01
	unstained		0				
	HBlue		1.54	-4.09/7.17			
	NRed		-12.79	-18.42/-7.16			
Time post-staining					2,152	126.29	<0.01
		24	0				
		48	-6.49	-12.12/-0.86			
		72	-25.67	-31.30/-20.04			
Staining treatment x time post-staining					4,152	2.92	0.023
	unstained	24	0				
	unstained	48	0				
	unstained	72	0				
	HBlue	24	0				
	HBlue	48	0.83	-7.13/8.79			
	HBlue	72	6.24	-1.72/14.20			
	NRed	24	0				
	NRed	48	-7.90	-15.86/0.06			
	NRed	72	-6.40	-14.36/1.55			
**Random effect**			**Estimate**	**SE**	**z**	**P**	
Ejaculate ID			70.22	25.73	2.73	<0.01	
**Marginal means**			**Mean**	**95% CI**			
Staining treatment x time post-staining							
	unstained	24	77.54	72.11/82.98			
	unstained	48	71.06	65.62/76.49			
	unstained	72	51.88	46.44/57.31			
	HBlue	24	79.09	73.65/84.52			

Journal of Experimental Biology • Supplementary information

	HBlue	48	73.43	68.00/78.87
	HBlue	72	59.66	54.22/65.09
	NRed	24	64.76	59.32/70.19
	NRed	48	50.37	44.94/55.81
	NRed	72	32.68	27.25/38.12

VCL (µm/s)

**Fixed effects**			**Estimate**	**95% CI**	**df**	**F**	**p**
Staining treatment					2,152	67.17	<0.01
	unstained		0				
	HBlue		0.46	-1.86/2.78			
	NRed		-8.26	-10.58/-5.94			
Time post-staining					2,152	146.48	<0.01
		24	0				
		48	-7.16	-9.48/-4.84			
		72	-13.52	-15.84/-11.20			
Staining treatment x time post-staining					4,152	1.92	0.11
	unstained	24	0				
	unstained	48	0				
	unstained	72	0				
	HBlue	24	0				
	HBlue	48	1.02	-2.27/4.30			
	HBlue	72	1.32	-1.96/4.60			
	NRed	24	0				
	NRed	48	2.03	-1.25/5.32			
	NRed	72	4.44	1.15/7.72			
**Random effect**			**Estimate**	**SE**	**z**	**P**	
Ejaculate ID			16.12	5.73	2.81	<0.01	
**Marginal means**			**Mean**	**95% CI**			
Staining treatment							
	Unstained		27.46	25.45/29.47			
	HBlue		28.70	26.69/30.71			
	NRed		21.36	19.35/23.37			
Timing post-insemination							
		24	31.76	29.75/33.77			
		48	25.61	23.60/27.62			
		72	20.15	18.14/22.16			

Journal of Experimental Biology • Supplementary information

Table S4. Effect of MRed staining on *in vitro* sperm motility and VCL after 20 minutes of incubation. We report the output of general linear mixed models with a normal distribution of errors where the staining treatment was included as a fixed effect and the ejaculate ID as a random intercept (because sperm from the same ejaculate were used for the two staining treatments). For the fixed effect, we report the degrees of freedom, the F and p values. For the random effect, we report the estimate with the standard error, the z and p values. We also report the marginal means with the 95% confidence intervals and the difference of marginal means between staining treatments with the adjusted 95% confidence intervals, t values and Bonferroni adjusted p values. All values were rounded to the second decimal place at the request of an anonymous referee. N = 17 ejaculates and 34 observations for each model.

Total motility (%)

**Fixed effect**				**df**	**F**	**p**
Staining treatment				1,16	0.42	0.52
**Random effect**		**Estimate**	**SE**	**z**	**p**	
Ejaculate ID		27.25	16.17	1.69	0.05	
**Marginal means**		**Mean**	**95% CI**			
Staining treatment						
	unstained	82.51	78.52/86.51			
	MRed	81.24	77.29/85.20			
**Difference of marginal means**		**Estimate**	**95% CI**	**t**	**p**	
Staining treatment						
	MRed - unstained	-1.27	-5.42/2.88	-0.65	0.52	

VCL (µm/s)

**Fixed effect**				**df**	**F**	**p**
Staining treatment				1,16	0.00	0.98
**Random effect**		**Estimate**	**SE**	**z**	**p**	
Ejaculate ID		6.87	5.46	1.26	0.10	
**Marginal means**		**Estimate**	**95% CI**			
Staining treatment						
	unstained	44.32	41.96/46.68			
	MRed	44.30	41.96/46.63			
**Differences of marginal means**		**Estimate**	**95% CI**	**t**	**p**	
Staining treatment						
	MRed - unstained	-0.03	-2.75/2.70	-0.02	0.98	

Table S5. Effect of MRed staining on *in vitro* sperm motility and VCL over time, up to 72 hours post-staining. We report the output of general linear mixed models with a normal distribution of errors where the staining treatment, the time post-staining (hours) and the two-way interaction were included as fixed effects and the ejaculate ID as a random intercept (because sperm from the same ejaculate were used for the two staining treatments). For the fixed effects, we report the parameter estimates with the 95% confidence intervals, the degrees of freedom, the F and p values. For the random effect, we report the parameter estimate with the standard error, the z and p values. We also report the marginal means with the 95% confidence intervals for the combination of staining treatment x timing post-insemination since the two-way interactions were statistically significant. All values were rounded to the second decimal place at the request of an anonymous referee. N = 17 ejaculates and 102 observations for each model.

Total motility (%)

**Fixed effects**			**Estimate**	**95% CI**	**df**	**F**	**p**
Staining treatment					1,80	15.55	<0.01
	unstained		0				
	MRed		-0.47	-5.59/4.65			
Time post-staining					2,80	56.37	<0.01
		24	0				
		48	0.15	-4.97/5.28			
		72	-14.95	-20.07/-9.82			
Staining treatment x time post-staining					2,80	3.43	0.04
	unstained	24	0				
	unstained	48	0				
	unstained	72	0				
	MRed	24	0				
	MRed	48	-9.07	-16.31/-1.82			
	MRed	72	-7.11	-14.35/0.14			
**Random effect**			**Estimate**	**SE**	**z**	**P**	
Ejaculate ID			116.41	44.51	2.62	<0.01	
**Marginal means**			**Mean**	**95% CI**			
Staining treatment x time post-staining							
	unstained	24	77.17	70.82/83.51			
	unstained	48	77.32	70.98/83.67			
	unstained	72	62.22	55.88/68.57			
	MRed	24	76.70	70.35/83.04			
	MRed	48	67.79	61.44/74.13			
	MRed	72	54.64	48.30/60.99			

Journal of Experimental Biology • Supplementary information

VCL (μm/s)

**Fixed effects**			**Estimate**	**95% CI**	**df**	**F**	**p**
Staining treatment					1,80	33.95	<0.01
	unstained		0				
	MRed		-2.71	-5.05/-0.36			
Time post-staining					2,80	127.03	<0.01
		24	0				
		48	-1.67	-4.02/0.67			
		72	-13.14	-15.48/-10.80			
Staining treatment x time post-staining					2,80	4.82	0.01
	unstained	24	0				
	unstained	48	0				
	unstained	72	0				
	MRed	24	0				
	MRed	48	-4.23	-7.54/-0.91			
	MRed	72	0.47	-2.84/3.78			
**Random effect**			**Estimate**	**SE**	**z**	**P**	
Ejaculate ID			18.51	7.24	2.55	<0.01	
**Marginal means**			**Mean**	**95% CI**			
Staining treatment x time post-staining							
	unstained	24	36.44	33.78/39.09			
	unstained	48	34.76	32.11/37.42			
	unstained	72	23.30	20.64/25.96			
	MRed	24	33.73	31.07/36.39			
	MRed	48	27.83	25.18/30.49			
	MRed	72	21.06	18.40/23.72			

Journal of Experimental Biology • Supplementary information

Table S6. Effect of time post-insemination on the number of sperm counted in the SSTs. We report the output of a generalized linear mixed model with a negative binomial distribution of errors. The model included the timing post-insemination (hours) as a fixed effect and the female identity as a random intercept (because sperm were counted in multiple SSTs per female). For the fixed effect, we report the degrees of freedom, the F and p values. For the random effect, we report the estimate with the standard error, the z and p values. We also report the marginal means with the 95% confidence intervals (on the original scale) and the difference of marginal means (on the link scale) between timings post-insemination with the adjusted 95% confidence intervals, t and Bonferroni adjusted p values. The Pearson c^2/df of the model = 1.30. All values were rounded to the second decimal place at the request of an anonymous referee. N = 9 females and 657 observations.

**Fixed effect**				**df**	**F**	**p**
Time post-insemination				2,648	0.71	0.49
**Random effect**		**Estimate**	**SE**	**z**	**p**	
Female ID		0.56	0.34	1.61	0.05	
**Marginal means on the original scale**		**Mean**	**95% CI**			
Time post-insemination						
	24	15.21	6.44/35.92			
	48	10.57	4.49/24.91			
	72	22.17	9.11/53.99			
**Difference of marginal means on the link scale**		**Estimate**	**95% CI**	**t**	**p**	
Time post-insemination						
	48 - 24	-0.36	-1.85/1.12	-0.59	1.00	
	72 - 24	0.38	-1.15/1.91	0.59	1.00	
	48 - 72	-0.74	-2.24/0.76	-1.19	0.71	

Table S7. Effect of staining treatment on the number of sperm counted in the SSTs at 48 hours post-insemination with a mix of HBlue and MRed sperm. We report the output of a generalized linear mixed model with a negative binomial distribution of errors. The model included the staining treatment as a fixed effect, the female identity and the SST identity nested within the female identity as random intercepts (because HBlue and MRed sperm were counted in the same SSTs and multiple SSTs were screened per female). For the fixed effect, we report the degrees of freedom, the F and p values. For the random effects, we report the estimates with the standard errors, the z and p values. We also report the marginal means with the 95% confidence intervals (on the original scale) and the difference of marginal means (on the link scale) between staining treatments with the adjusted 95% confidence intervals, t and Bonferroni adjusted p values. The Pearson χ^2/df of the model = 1.34. All values were rounded to the second decimal place at the request of an anonymous referee. N = 3 females, 100 tubules, and 600 observations.

**Fixed effect**				**df**	**F**	**p**
Staining treatment				1,569	52.41	<0.01
**Random effects**		**Estimate**	**SE**	**z**	**p**	
Female ID		0.11	0.09	1.25	0.11	
SST ID * Female ID		0.03	0.02	1.48	0.07	
**Marginal means on the original scale**		**Mean**	**95% CI**			
Staining treatment						
	HBlue	10.64	7.14/15.85			
	MRed	5.97	4.00/8.90			
**Difference of marginal means on the link scale**		**Estimate**	**95% CI**	**t**	**p**	
Staining treatment						
	HBlue - MRed	0.58	0.42/0.74	7.24	<0.01	

Journal of Experimental Biology • Supplementary information

Table S8. Effect of staining treatment and insemination order on the number of sperm counted in the SSTs following two sequential inseminations with HBlue or MRed sperm. We report the output of a generalized linear mixed model with a negative binomial distribution of errors. The model included the staining treatment, the order of the inseminations and the two-way interaction as fixed effects, the female identity and the SST identity nested within the female identity as random intercepts (because HBlue and MRed sperm were counted in the same SSTs and multiple SSTs were screened per female). For the fixed effects, we report the degrees of freedom, the F and p values. For the random effects, we report the estimates with the standard errors, the z and p values. We also report the marginal means with the 95% confidence intervals (on the original scale) and the difference of marginal means (on the link scale) between staining treatments and insemination order with the adjusted 95% confidence intervals, t and Bonferroni adjusted p values. The Pearson c^2/df of the model = 0.76. All values were rounded to the second decimal place at the request of an anonymous referee. N = 10 females, 617 SSTs, and 1234 observations.

**Fixed effects**					**df**	**F**	**p**
Staining treatment					1,615	247.44	<0.01
Insemination order					1,615	904.83	<0.01
Staining treatment x insemination order					1,615	0.00	0.96
**Random effects**			**Estimate**	**SE**	**z**	**p**	
Female ID			0.13	0.06	2.08	0.02	
SST ID * Female ID			0.29	0.04	7.36	<0.01	
**Marginal means on the original scale**			**Mean**	**95% CI**			
Staining treatment							
	HBlue		21.43	16.78/27.38			
	MRed		9.29	7.24/11.90			
Insemination order							
		first	6.38	4.98/8.17			
		last	31.18	24.40/39.86			
**Difference of marginal means on the link scale**			**Estimate**	**95% CI**	**t**	**p**	
Staining treatment							
	HBlue - MRed		0.84	0.73/0.94	15.73	<0.01	
Insemination order							
		last - first	1.59	1.48/1.69	30.08	<0.01	

Journal of Experimental Biology • Supplementary information

Table S9. Effect of staining treatment and insemination order on the distance (µm) of sperm from the bottom of the SSTs following two sequential inseminations with HBlue or MRed sperm. We report the output of a general linear mixed model with a normal distribution of errors. The model included the staining treatment, the order of the inseminations and the two-way interaction as fixed effects, the female identity and the SST identity nested within the female identity as random intercepts (because the distance of multiple sperm was measured per SST and multiple SSTs were screened per female). For the fixed effects, we report the degrees of freedom, the F and p values. For the random effects, we report the estimates with the standard errors, the z and p values. We also report the marginal means with the 95% confidence intervals and the difference of marginal means between staining treatments and insemination order with the adjusted 95% confidence intervals, t and Bonferroni adjusted p values. All values were rounded to the second decimal place at the request of an anonymous referee. N = 10 females, 96 tubules, and 3877 observations.

**Fixed effects**					**df**	**F**	**p**
Staining treatment					1,3778	1.18	0.28
Insemination order					1,3778	45.23	<0.01
Staining treatment x insemination order					1,3778	0.39	0.53
**Random effects**			**Estimate**	**SE**	**z**	**p**	
Female ID			37.54	36.14	1.04	0.15	
SST ID * Female ID			290.96	49.30	5.90	<0.01	
**Marginal means**			**Mean**	**95% CI**			
Staining treatment							
	HBlue		53.32	47.94/58.69			
	MRed		52.05	46.62/57.47			
Insemination order							
		first	56.61	51.11/62.11			
		last	48.75	43.45/54.05			
**Difference of marginal means**			**Estimate**	**95% CI**	**t**	**p**	
Staining treatment							
	HBlue - MRed		1.27	-1.02/3.56	1.09	0.28	
Insemination order							
		last - first	-7.86	-10.15/-5.57	-6.73	<0.01	

Table S10. Effect of staining treatment on the distance (µm) of sperm from the bottom of the SSTs following a simultaneous insemination with HBlue and MRed sperm. We report the output of a general linear mixed model with a normal distribution of errors. The model included the staining treatment as fixed effect, the female identity and the SST identity nested within the female identity as random intercepts (because the distance of multiple sperm was measured per SST and multiple SSTs were screened per female). For the fixed effect, we report the degrees of freedom, the F and p values. For the random effects, we report the estimates with the standard errors, the z and p values. We also report the marginal means with the 95% confidence intervals and the difference of marginal means between staining treatments with the adjusted 95% confidence intervals, t and Bonferroni adjusted p values. All values were rounded to the second decimal place at the request of an anonymous referee. N = 3 females, 30 tubules, and 980 observations.

**Fixed effect**				**df**	**F**	**p**
Staining treatment				1,949	0.04	0.85
**Random effects**		**Estimate**	**SE**	**z**	**p**	
Female ID		0				
SST ID * Female ID		130.53	40.65	3.21	<0.01	
**Marginal means**		**Mean**	**95% CI**			
Staining treatment						
	HBlue	38.92	34.31/43.52			
	MRed	39.22	34.41/44.03			
**Difference of marginal means**		**Estimate**	**95% CI**		**t**	**p**
Staining treatment						
	HBlue - MRed	-0.30	-3.46/2.86		-0.19	0.85
